# Archaean oxygen oases driven by pulses of enhanced phosphorus recycling in the ocean

**DOI:** 10.1038/s41561-025-01678-4

**Published:** 2025-04-16

**Authors:** Fuencisla Cañadas, Romain Guilbaud, Philip Fralick, Yijun Xiong, Simon W. Poulton, Maria-Paz Martin-Redondo, Alberto G. Fairén

**Affiliations:** 1 Centro de Astrobiología (CAB), CSIC-INTA, Madrid, Spain; 2 Géosciences Environnement Toulouse, UMR 5563 CNRS, Toulouse, France; 3 Department of Geology, Lakehead University, Thunder Bay, Ontario Canada; 4 School of Earth and Environment, University of Leeds, Leeds, UK; 5 Department of Astronomy, Cornell University, Ithaca, NY USA

**Keywords:** Geochemistry, Biogeochemistry

## Abstract

Earth’s first rise in atmospheric oxygen between about 2.43 billion and 2.1 billion years ago fundamentally transformed the atmosphere and oceans, setting the foundation for the evolution of complex life. However, geochemical evidence reveals intermittent oceanic oxygen oases before the rise of atmospheric oxygen, although the mechanisms that drove the production and accumulation of oxygen remain poorly constrained. Here we present redox-sensitive trace metal and iron speciation data, and phosphorus phase partitioning results, for a 2.93-billion-year-old drill core from the Red Lake area, Canada, to reconstruct oceanic phosphorus cycling and links to oxygen production in the dominantly anoxic, iron-rich Archaean ocean. Our data document one of the earliest known intervals of surface water oxygen accumulation, predating the first accumulation of atmospheric oxygen by about 500 Ma. These intervals were preceded by ferruginous intervals and intervals of enhanced sulfide availability, which led to pulsed increases in oceanic phosphorus bioavailability via anoxic recycling from sediments. Enhanced phosphorus bioavailability would have helped stimulate photosynthetic primary productivity and organic carbon burial, probably exerting a major control on the episodic development of oxygen oases in the late Archaean ocean. This, in turn, led to a critical transitional phase in the development of an oxygenated surface environment.

## Abstract (web summary)

Periods with enhanced iron and sulfide availability that promoted recycling of bioavailable phosphorus from sediments contributed to episodic development of oxygen oases in the Archaean ocean, according to analysis of trace metals, phosphorus and iron from 2.9-billion-year-old sediments.

## Main

The transition to a persistently oxygenated atmosphere during the Great Oxidation Event (GOE) ~2.43‒2.1 billion years ago (Ga)^1^ has ultimately been attributed to the evolution of oxygenic photosynthesis^2^. Cyanobacterial ancestors were among the first organisms to perform oxygenic photosynthesis, and initially, the oxygen they produced would have reacted with reduced species in the ocean and atmosphere, thereby preventing atmospheric accumulation. However, local oxygen accumulation in oceanic oxygen oases and transient ‘whiffs’^3–5^ of atmospheric oxygen before the GOE, representing oxygen production but not atmospheric accumulation^6^, appear to have been a crucial transitional step in the progression towards persistent Earth surface oxygenation^7^.

Evidence of pre-GOE whiffs of oxygen is substantiated by multiple independent geochemical proxies, including molybdenum and rhenium enrichments at ~2.5–2.6 Ga in South Africa^8^ and ~2.5 Ga in Western Australia^3^, manganese oxides burial and molybdenum stable isotope compositions at ~2.5 Ga (ref. ^9^) in Australia and ~2.95 Ga (refs. ^10,11^) in South Africa, mobilization of selenium by free oxygen at ~2.5 Ga (ref. ^12^) in Australia and Ce abundance anomalies at ~2.6 Ga (ref. ^13^) in South Africa and Canada. In the Red Lake area (Canada), the presence of whiffs of oxygen has been inferred in 2.93-Ga deposits based on trace element enrichments and rare earth elements^4,5^. However, the mechanisms that promoted the production and accumulation of oxygen have scarcely been explored, reflecting a critical knowledge gap in our understanding of oxygen evolution on the early Earth. Furthermore, the factors causing the delay in pervasive oxygenation of the atmosphere–ocean system after the establishment of oxygenic photosynthesis in the Archaean remain poorly constrained, particularly regarding the role of bio-limiting nutrients such as nitrogen and phosphorus (P)^14^.

On geologic timescales, P is generally considered the ultimate limiting nutrient for primary productivity, which is intrinsically linked to organic carbon (C_org_) production and burial, and therefore to atmospheric oxygen production^15^. Multiple studies point to persistent low seawater P concentrations throughout the Archaean^16,17^ and Proterozoic^18,19^, with the depletion in P primarily attributed to scavenging by iron minerals in the water column^17–19^. However, recent studies have challenged the view of low P in the Archaean ocean^20,21^, and while there is evidence for P–C_org_–O_2_ coupling in late Neoarchaean marine sediments deposited immediately before the GOE^7^, the behaviour of the P cycle in earlier oxygen oasis settings remains entirely unconstrained.

Here we seek to bridge this knowledge gap by conducting an in-depth analysis of P cycling in Mesoarchaean (~2.93 Ga) open marine sediments from the Ball Assemblage, Canada, to evaluate potential links to periodic oxygenation of the early Earth’s surface. We combine P phase partitioning with Fe speciation and redox-sensitive trace metal data to evaluate local redox controls on P cycling and its availability. This constitutes the oldest examination of coupled redox and P cycling in marine sediments, reflecting one of the earliest known examples of oasis-style oceanic oxygenation.

## Geological setting

To investigate local redox conditions, P bioavailability and its role in primary productivity, C_org_ burial and subsequent oxygen production, we studied a Mesoarchaean drill core (NGI10-31) from the Red Lake Greenstone Belt, Canada (Supplementary Fig. 1). The Red Lake area represents the oldest carbonate platform on Earth^4,5,22^. It was deposited in an open marine setting and contains some of the earliest geochemical evidence for oxygenic photosynthesis and whiffs of oxygen^5,23^. The NGI10-31 core consists of alternating siliciclastics and offshore chemical sediments, which are comprised of chert, alongside oxide-rich and sulfide-rich iron formations from the basinal facies of the Red Lake carbonate platform^24^ (Supplementary Information provides full details of the geologic setting, age constraints, core location and evaluation of metamorphism).

## Ocean redox conditions

To constrain water column redox variability through the NGI10-31 core, we combine Fe speciation with redox-sensitive trace metal (Mo, V and U) systematics (Supplementary Information provides detailed analytical procedures and Methods provides the interpretational framework). To evaluate trace metal data, we calculate enrichment factors (EFs), utilizing a modified approach^25,26^ to allow for artificially inflated EF values in chemical sediments (iron formations and cherts)^25^, due to their low Al content (Methods).

**Fig. 1:**
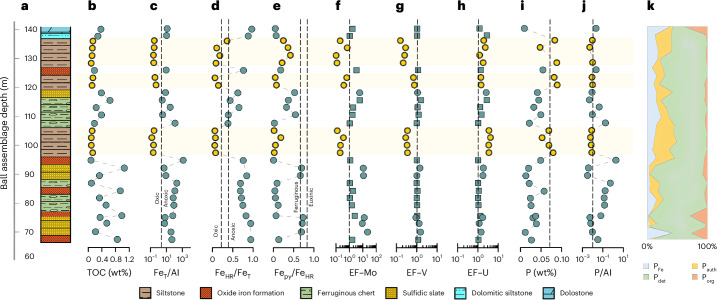
Stratigraphy and geochemistry of the NGI10-31 drill core. **a**, Stratigraphy and lithology of the NGI core. **b**, Total organic carbon contents. **c**, Total iron versus aluminium ratio reported as wt%/wt%. **d**, Highly reactive iron versus total iron. **e**, Pyrite–Fe versus highly reactive Fe. **f**–**h**, Enrichment factors (EFs) for molybdenum (**f**), vanadium (**g**) and uranium (**h**). **i**, Total phosphorus content. **j**, Phosphorus versus aluminium ratio reported as wt%/wt%. **k**, Proportion of P_det_, P_auth_, P_org_ and P_Fe_ within the total P pool. The yellow dots and light-yellow areas indicate the oxic water column conditions. In **f**–**h**, circle symbols correspond to EFs calculated with the standard Al normalization equation, and square symbols represent revised EFs calculations for chemical samples (Methods). Dashed lines: in **c**, upper boundary for recognition of anoxic deposition; in **d**, boundaries for identifying oxic (<0.22) and anoxic deposition (>0.38), with equivocal samples falling between these lines; in **e**, boundaries for identifying euxinic (>0.8) from ferruginous (<0.6) deposition with possibly euxinic conditions in between 0.6 and 0.8; in **c**, **i** and **j**, represent average shale values (ref. ^46^). Note that samples NGI 26 and NGI 29 have been excluded from **i**, **j** and **k** (see ‘Phosphorus phase partitioning’ section in the Supplementary Information).

Generally elevated Fe_HR_/Fe_T_ (>0.38) and Fe_T_/Al (>0.66) ratios, combined with enrichments in Mo and V, indicate deposition under dominantly anoxic bottom water conditions^27,28^, but with better oxygenated intervals potentially being indicated by lower values for these proxies during deposition of siltstones (Fig. 1; below). However, the U record is relatively stable, with little evidence for sediment enrichment. In the Red Lake area, redox-sensitive metals were delivered to seawater largely via oxidative continental weathering^5^. We thus attribute the limited enrichment in U to a particularly low oxidative weathering influx, relative to Mo and V, due to the high oxidation requirements of minerals such as uraninite, the main mineral host for crustal U, relative to Mo and V host phases^29^. Intervals of water column anoxia are further supported by moderate EF–Mo values (>1 to <10; Fig. 1), which commonly occur via a particulate shuttle mechanism following Mo uptake by Fe minerals precipitating in a ferruginous water column^26,30^.

We note, however, that because the apparent development of better oxygenated water column conditions is restricted to siltstone intervals, this may represent rapid sedimentation due to relocation of the river system, which would potentially mask water column Fe_HR_/Fe_T_ and Fe_T_/Al enrichments even under anoxic conditions^31^. Conversely, it is well documented that in modern alluvial systems (for example, estuaries or river input systems), high Fe_HR_ inputs can lead to high Fe_HR_/Fe_T_ ratios, giving an apparent anoxic depositional condition^32,33^, especially if eroded sediments are sourced from highly weatherable mafic volcanic terrains, such as in the Red Lake area. However, the low Fe_HR_/Fe_T_ and Fe_T_/Al ratios during deposition of the siltstone horizons (Fig. 1) rule out this second possibility.

In assessing the possibility that Fe_HR_/Fe_T_ and Fe_T_/Al ratios were masked by rapid sedimentation, we note that EFs for Mo and V are similarly depleted across these intervals (Fig. 1f,g). The relative depletion in Mo may also be an expectation during rapid sedimentation under ferruginous conditions because this would potentially mask the drawdown of Mo delivered to the sediment via the particulate shuttle mechanism. However, the geochemical behaviour of V contrasts with that of Mo in that it is primarily affected by diagenetic transformations close to the sediment–water interface (Methods). Indeed, under partially oxygenated (dysoxic) conditions, V may be mobilized and lost from the sediment^34^. Rapid sedimentation would probably minimize this loss of V to the overlying water column, which may have occurred across the middle siltstone horizon, where V is not depleted (Fig. 1g). However, while the redox interpretation for the siltstone layers is complicated by the potential for rapid sedimentation, the depletion in V observed across two of these horizons is consistent with loss of V from the sediments under dysoxic conditions. This is similar to the redox state proposed for the 2.6–2.5-Ga Campbellrand–Malmani carbonate platform in South Africa^8^, and we note that partially oxygenated surface waters may have dominated during the early stages of biospheric oxygenation.

Indeed, the presence of at least partially oxygenated surface waters in the vicinity of a redox boundary separating the stromatolite-rich shallow shelf from further offshore lithofacies has been invoked as an oxidative mechanism for the deposition of the oxide iron formations (IFs) in the Red Lake^5,23^ and nearby areas^4,22^. Similarly, oxidation and removal of Ce and Mn from seawater and subsequent enrichment of these elements in chemical sediments near the carbonate platform required oxygen in the depositional environment^5^. Thus, independent lines of evidence suggest that the water column periodically became partially oxygenated in the Red Lake area, with the extent of oxygenation progressively increasing in time and space in the run-up to the GOE^9,35^.

In addition, episodes of enhanced sulfide availability, at least in shallow pore waters but also potentially in the water column, are implied for three intervals (at 67–75 m, 88–93 m and 118 m) by elevated Fe_HR_/Fe_T_ (>0.38) and high Fe_Py_/Fe_HR_ (>0.6) ratios, along with elevated EF–Mo (>6) and EF–V (>1) values. The consensus for Precambrian sedimentary sulfide production invokes microbial reduction of sulfate, itself derived from oxidative weathering and continental run-off^36^. Therefore, sulfide production in our ~2.93-Ga sediments implies oxidative weathering of Archaean^37^ continents and supply of riverine sulfate. Alternatively, as supported by rare earth element patterns^12,27^, seawater sulfate could have been sourced from hydrothermally derived upwelling waters.

## Phosphorus cycling in the dominantly iron-rich Archaean ocean

The transfer of P to marine sediments primarily occurs through the deposition of detrital apatite, organic matter and Fe minerals. In the modern ocean, rivers provide the dominant source of dissolved P (ref. ^38^), and concentrations can be sustained in the water column by the remineralization of sinking organic matter. Indeed, under oxic conditions, most organic P is remineralized during deposition, whereas a considerable fraction of organic and Fe-bound P is released to the pore waters of anoxic sediments during diagenesis^39^. Under oxic water column conditions, P released to pore waters during early diagenesis is dominantly trapped in the sediment via ‘sink switching’, either forming carbonate fluorapatite or re-adsorbing to Fe minerals near the sediment–water interface^40,41^. However, under euxinic water column conditions or under ferruginous conditions where pore waters are sulfidic close to the sediment–water interface, a major proportion of the P released during diagenesis may be recycled back to the water column, potentially promoting a positive productivity feedback^7,42^. By contrast, under oligotrophic ferruginous conditions, P adsorbed to Fe (oxyhydr)oxide minerals formed in the water column may be efficiently retained in the sediment^7,18^. In addition, under anoxic conditions, P is preferentially released from organic matter during remineralization, particularly during microbial sulfate reduction^42,43^. This results in elevated molar C_org_/P_org_ ratios, surpassing the canonical Redfield ratio of 106/1 (ref. ^44^). In the Archaean, therefore, the nature of local redox conditions, both in the water column and in the sediment, would have exerted a strong control on the fate of P.

To evaluate the P cycle on the Red Lake Platform, we determined the phase partitioning of P via a sequential extraction scheme adapted to ancient sediments^45^ (Methods). The technique targets four operationally defined P pools, including iron-bound P (P_Fe_), authigenic P (P_auth_), organic-bound P (P_org_) and detrital P (P_det_), with the sum of P_Fe_, P_auth_ and P_org_ defining a reactive P pool (P_reac_), representing P that is potentially bioavailable during deposition and early diagenesis. The correlation between P_det_ and unreactive detrital elements such as Ti in our samples (Supplementary Fig. 3) implies that P_det_ is dominantly derived from detrital sources and not from diagenetic recrystallization of P_auth_ (refs. ^18,45^) (Methods and Supplementary Information provide further discussion).

Total P contents in the NGI10-31 core are generally below the average shale value^46^ (Fig. 1i), which is consistent with a low supply of P from the water column due to global depletion of P via widespread removal in association with Fe minerals under ferruginous conditions^18,19^. Accordingly, P_det_ is the largest P pool, due to the dominance of detrital riverine inputs over bioavailable forms (Fig. 1k). The two sections of the core that capture deposition under oxic water column conditions exhibit P contents close to average shale (Fig. 1i,j). These intervals also record the highest P_reac_ values, dominantly characterized by P_Fe_ and P_auth_ phases (Fig. 1k), with the latter resulting from diagenetic sink switching from phases (P_Fe_ and P_org_) that dominantly reflect drawdown of dissolved water column phosphate^47^. At the bottom of the core (75 to 90 m) and immediately after the lower oxic interval (108 to 112 m), P/Al surpasses the average shale value, but this is probably due to the very low Al contents in iron formations and ferruginous chert, and in both cases, P_det_ represents ~70% of the total P budget.

## Archaean oxygen oases within a P-limited ferruginous ocean

**Fig. 2:**
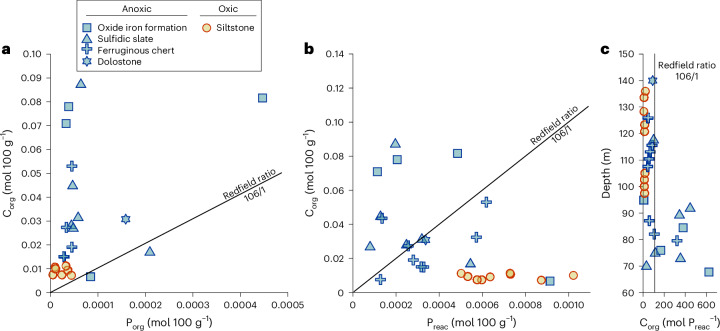
Relationships between C_org_ and the P partitioning in the NGI10-31 core. **a**, C_org_ versus P_org_ in moles per 100 g of sediment. **b**, C_org_ versus P_reac_ in moles per 100 g of sediment. **c**, Depth (m) versus C_org_/P_reac_ ratios. Black lines represent the Redfield ratio (106/1).

We next explore C/P ratios through the NGI10-31 core to address potential P limitation on primary productivity. Most of the core exhibits elevated C_org_/P_org_ ratios, approaching ~2,000 (Fig. 2a), considerably above the Redfield ratio of 106/1 (ref. ^44^). Such elevated C_org_/P_org_ ratios demonstrate pronounced preferential release of P during anaerobic remineralization of organic matter under anoxic conditions^39,43^. This P release occurred during intervals of enhanced sulfide availability (high Fe_HR_/Fe_T_ and Fe_Py_/Fe_HR_ ratios; Fig. 1d,e and Supplementary Fig. 4), but also during ferruginous intervals (that is, deposition of oxide IFs and, to a lesser extent, dolostone and ferruginous cherts; Fig. 2a), where there was probably limited sulfide generation (as evidenced by low Fe_Py_/Fe_HR_ ratios; Fig. 1e). This highlights that while microbial sulfate reduction is important for driving preferential release of P during organic matter remineralization^42,43^, other microbial pathways (such as dissimilatory Fe reduction) may also promote preferential P release. Indeed, the organic matter preserved in these ferruginous, low-sulfide samples is relatively high (between 0.54 wt% and 1.05 wt%; Fig. 1b), suggesting abundant availability to fuel microbial remineralization pathways.

To address whether the preferential release of P from organic matter during diagenesis resulted in P recycling to the water column, we next consider C_org_/P_reac_ ratios (Fig. 2b). Except for two samples, sulfidic slates and oxide-rich IFs mostly plot well above the Redfield ratio, confirming efficient recycling of P to the water column during early diagenesis. Samples showing no evidence for P recycling (hence plotting below the Redfield ratio) are less provisioned in organic matter, with total organic carbon (TOC) contents <0.5 wt%, suggesting that the supply of organic carbon exerted a positive feedback on P recycling under anoxic conditions. Contrasting with relatively TOC-rich samples (that is, >0.5 wt% TOC), ferruginous cherts and dolostone also mostly yield C_org_/P_reac_ plotting near or below the Redfield ratio. This supports limited P recycling to the water column, with P fixation as P_auth_ and P_Fe_ for the less productive, carbonate- or chert-dominated facies. Samples deposited under oxic water conditions have C_org_/P_reac_ ratios below the Redfield ratio (Fig. 2b). This is consistent with efficient P fixation in the sediment after drawdown in association with organic matter and, to a lesser extent, Fe minerals, coupled with extensive release of P during aerobic organic matter oxidation and trapping of that P by microbial biomass^48^. If organic matter was the main carrier of reactive phosphorus under oxic water column conditions, this suggests that P released from organic decay was effectively trapped, ultimately as P_auth_ or P_Fe_ (Fig. 1k). This process is reflected by the increase in P_reac_ (Fig. 1k) and P/Al ratios close to the average shale^41^ value (Fig. 1j).

Archaean oxygen oases were probably spatially and temporally limited, and their development was largely dependent on local redox conditions and nutrient cycling. A comparison between our C_org_/P_reac_ results and data from Neoarchaean (2.65 to 2.43 Ga) S- and Fe-rich samples from South Africa^7^ suggests that oxygen oases were less extensive and less intense ~2.93 Ga, with average Neoarchaean C_org_/P_reac_ values of 3,900 ± 5,764, indicating particularly active P recycling^7^, in sharp contrast to average values of 128 ± 160 in our earlier Archaean samples (Fig. 2c). This aligns with a period of increased sulfate delivery from 2.8 to 2.4 Ga (refs. ^49,50^), which would have enhanced sulfide production and, consequently, the extent of P recycling. Enhanced P recycling probably created a positive productivity feedback that progressively increased oxygen production in these oasis-style settings. Over time, oxygenation expanded and intensified, progressing from limited areas in the Archaean to well-oxygenated continental shelves by ~2.5 Ga (ref. ^9^). Ultimately, this led to coupled atmosphere–ocean oxygenation by 2.32 Ga (ref. ^35^), when oxygen sources began to surpass oxygen sinks, driving Earth’s surface oxygenation^1^.

**Fig. 3:**
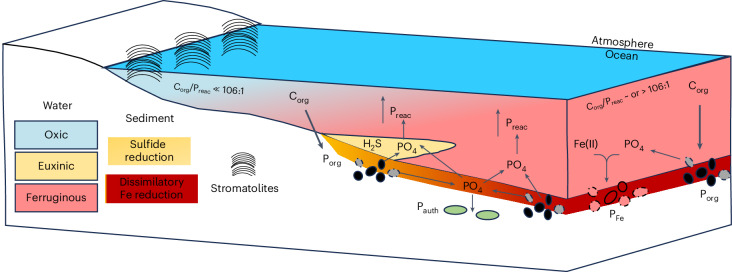
Conceptual model. The development of oxygen oases was strongly localized and limited by nutrient availability, particularly phosphorus. Ferruginous settings typically acted as phosphorus sinks under oligotrophic conditions, restricting productivity and the potential for oxygen production. However, during enhanced microbial iron reduction or when sulfidic intervals developed near the sediment–water interface, efficient phosphorus recycling could fuel localized primary production and drive oxygen generation. P recycling during periods of enhanced sulfide availability triggered additional P recycling from the IFs deposited deeper in the water column. P commonly underwent efficient recycling to the water column through anaerobic remineralization of organic matter and reduction of iron (oxyhydr)oxides, resulting in C_org_/P_reac_ ratios close to or higher than the Redfield ratio (106/1). The increased bioavailability of P promoted enhanced primary productivity, increased C_org_ burial and periodic accumulation of oxygen in surface waters.

Our results collectively depict a dynamic redox scenario that promoted P recycling into the water column. Figure 3 illustrates how P recycling was primarily driven by sulfide production, initially triggered by oxygen ‘whiffs’ that facilitated an influx of sulfate, promoting sulfide production. This process, in turn, helped sustain productivity and oxygen generation. Specifically, during periods of enhanced sulfide availability, sulfidic slates were deposited, enhancing P recycling back to the water column. The subsequent increase in organic matter generation triggered additional P recycling from the IFs deposited deeper in the water column (note that these IFs are relatively enriched in TOC, up to 1 wt%), beneath the sulfidic wedge. The extent of the sulfidic zone probably fluctuated over time, with its spatial distribution waxing and waning, leading to the alternating deposition of sulfidic slates, IF and ferruginous chert observed in the NGI10-31 core. Although Archaean seawater was generally P limited, and ferruginous sediments were typically P depleted and dominated by detrital inputs, microbially mediated P recycling to the water column appears to have been sufficient to stimulate photosynthetic primary production in the oxygen oasis of the Red Lake area, as evidenced by diverse stromatolitic assemblages^5,23^.

The cycling and recycling of P played a critical role in shaping Earth’s early biosphere and the evolution of life, particularly during the Archaean. Our findings highlight the dynamic redox conditions of the Archaean ocean, where specific redox conditions at the bottom of the water column—such as organic-rich ferruginous or euxinic environments—enabled the recycling of P from the sediments into the water column. In these relatively shallow water settings, recycled P would have provided an additional source of bioavailable phosphorus to fuel primary productivity, particularly in localized oxygen oases. These oases, though spatially and temporally limited, probably facilitated the development of early microbial life, including stromatolite-forming cyanobacteria, by maintaining a supply of bioavailable P. In turn, enhanced local organic carbon burial would have allowed for oxygen accumulation and the establishment of intermittent oxygen oases in shallow waters, marking a crucial transitional phase in Earth’s oxygenation history.

The interplay between redox conditions and nutrient cycling may have created feedback loops that promoted oxygen production in these localized environments, contributing to gradual increases in atmospheric and oceanic oxygen levels. This process, which could have extended over millions of years, set the stage for the eventual rise of more complex life forms and the evolution of Earth’s biogeochemical cycles, influencing the planet’s capacity to support life in the long term. Thus, our data support a biogeochemical coupling between bioavailable P, organic carbon and O_2_ production some 500 million years before the first accumulation of atmospheric O_2_. This suggests that the mechanisms linking the cycling of bioavailable nutrients to coevolving organisms, which led to the modern stoichiometry of life, may trace their origins back to the Archaean.

## Methods

### Iron speciation

The iron speciation method evaluates local redox conditions^51^. It targets four operationally defined iron fractions, including carbonate-associated iron (Fe_Carb_), ferric oxides (Fe_Ox_), magnetite (Fe_Mag_) and pyrite-associated Fe (Fe_Py_), which together comprise highly reactive iron (Fe_HR_)^31^. Ratios of Fe_HR_ to total Fe (Fe_T_) above 0.38 suggest deposition from anoxic bottom waters^52^. By contrast, Fe_HR_/Fe_T_ ratios below 0.22 suggest deposition from oxic bottom waters, whereas intermediate values are considered equivocal^52^. Shales and iron-formation samples deposited in ferruginous settings can be impacted by the transfer of non-sulfidized Fe_HR_ to poorly reactive sheet silicates during early diagenesis, which lowers Fe_HR_/Fe_T_ ratios^53,54^. To account for this possibility, we also considered Fe_T_/Al ratios, whereby values >0.66 provide a robust indication of anoxic depositional conditions^55^, and thus we identify anoxic water column deposition by a combination of Fe_HR_/Fe_T_ > 0.38 and/or Fe_T_/Al > 0.66 (Fig. 1).

### Redox-sensitive trace metals

Redox-sensitive elements such as V, Mo and U tend to be more soluble under oxidizing conditions and less soluble under reducing conditions, resulting in authigenic enrichments in oxygen-depleted sedimentary facies^27^. The removal of these metals from seawater under anoxic conditions may result in sediment enrichments several orders of magnitude higher than detrital values^27^.

Under oxic conditions, Mo is transported as the molybdate anion (MoO_4_^2−^) and remains largely unreactive, with water column drawdown primarily occurring via uptake to Fe–Mn (oxyhydr)oxide minerals^56^. However, particle-reactive thiomolybdate forms under sulfidic conditions, commonly resulting in extensive sequestration in the sediments^57,58^. Under oxic–suboxic conditions, U is present mainly in the form of uranyl carbonate (UO_2_(CO_3_)_3_^4−^) and is largely chemically unreactive^59^. However, under anoxic conditions in the sediments, U(VI) is reduced to U(IV), which may result in enrichments in the sediment, regardless of whether the water column is euxinic or ferruginous^60^. Vanadium is commonly transported to sediments as the vanadate ion (H_2_V(VI)O^−^_4_) adsorbed onto Mn oxides. Under mildly reducing conditions, where Mn oxides are reduced to Mn^2+^, V is commonly released from the sediment^34^, resulting in V depletion. Under anoxic conditions, vanadate is reduced to the vanadyl ion (V(IV)O_2_^+^), which is highly surface reactive and tends to be retained in the sediment^57^.

### Redox-sensitive trace metal enrichment factors

Redox-sensitive trace metals may be controlled by intrinsic basinal factors, such as provenance and thus they are generally normalized to aluminium to account for terrigenous detrital inputs. A common way to approach this normalization is via the calculation of enrichment factors (EFs) for a specific element relative to average continental crust^61^. It is important to highlight that the primary control on elemental ratios in siliciclastics is usually the source area composition^62^. Therefore, the composition of the source rocks must be taken into consideration when calculating trace metal EFs. In this case, we normalized to lower continental crust (LCC) average values^63^, which better represent the mafic and ultramafic composition of the Red Lake area rocks, via the formula:

$${{\rm{EF}}}_{{\rm{element}}}=({\rm{Elemen}}{\rm{t}}/{\rm{Al}})_{{\rm{sample}}}/({\rm{Element}}/{\rm{A}}{\rm{l}})_{{\rm{LCC}}}$$

Whereas this equation is valid for siliciclastic sediments (and is used for siliciclastic sediments in the present study), the application of EF values to chemical sediments is problematic, as elevated values are commonly obtained relative to siliciclastic sediments^27^ due to the low detrital Al component characteristic of chemical sediments. An alternative approach is to calculate excess trace metal contents^64^:

$${{\rm{Element}}}_{{\rm{excess}}}={{\rm{Element}}}_{{\rm{sample}}}-\left({{\rm{Al}}}_{{\rm{sample}}}\times \frac{{{\rm{Element}}}_{{\rm{LCC}}}}{{{\rm{Al}}}_{{\rm{LCC}}}}\right)$$

However, this approach lacks the utility of EF values as they provide no information on the relative degree of enrichment, and consideration alongside well-calibrated siliciclastic EF values is not possible. To address this, we adopt a recently modified approach^25,26^, which utilizes ‘excess’ trace metal concentrations and recasts these data as EF^*^ values:

$${{{\rm{Element}}}_{{\rm{EF}}}}^{* }=\frac{{{\rm{Element}}}_{{\rm{excess}}}+{{\rm{Element}}}_{{\rm{LCC}}}}{{{\rm{Element}}}_{{\rm{LCC}}}}$$

With this approach, EF^*^ values calculated for chemical sediments (including carbonates, cherts and IFs) can be directly compared to EF values calculated for siliciclastic sediments (Supplementary Table 1).

### Phosphorus phase partitioning

We performed a sequential P extraction scheme adapted for ancient sedimentary rocks^45^. The method targets four operationally defined P pools, including iron-bound P (P_Fe_), authigenic P (P_aut_), organic-bound P (P_org_) and crystalline apatite P (dominantly detrital P; P_det_). Reactive P (P_reac_) is considered to be potentially available to organisms and is calculated as the sum of P_Fe_ + P_auth_ + P_org_. P_det_ is considered unreactive during early diagenesis and is buried in the sediment without biogeochemical interactions.

## Online content

Any methods, additional references, Nature Portfolio reporting summaries, source data, extended data, supplementary information, acknowledgements, peer review information; details of author contributions and competing interests; and statements of data and code availability are available at 10.1038/s41561-025-01678-4.

## Supplementary information

Supplementary Information Geological context, methods, data quality checks, Supplementary Fig. 4 and references.

Supplementary Table 1 Supplementary Table 1.

## Data Availability

All data generated or analysed during this study are available via Figshare at 10.6084/m9.figshare.28359224 (ref. ^65^) and included within the published article and its Supplementary Information files.
