# Supplementary material for: Archaean oxygen oases driven by pulses of enhanced phosphorus recycling in the ocean
Source: Nat Geosci. 2025 Apr 16;18(5):430–5. doi: 10.1038/s41561-025-01678-4 (PMC12074997; doi:10.1038/s41561-025-01678-4)
Supplement: Supplementary file 1 — Geological context, methods, data quality checks, Supplementary Fig. 4 and references. [file 41561_2025_1678_MOESM1_ESM.pdf]

nature geoscience

Supplementary information https://doi.org/10.1038/s41561-025-01678-4

Archaean oxygen oases driven by pulses of enhanced phosphorus recycling in the ocean

In the format provided by the authors and unedited

This PDF file includes:

Supplementary Information

- Geological context
- Methods
- Data quality checks
- Supplementary Figure 4
- References

Supplementary Information

Geological context

The Red Lake carbonate platform was deposited in an open marine environment and lies in the Red Lake Greenstone Belt (RLGB) of the Uchi Subprovince in western Superior Craton (Supplementary Figure 1a). Supracrustal rocks of the Uchi Subprovince include Mesoarchean tholeiitic-komatiitic oceanic platform sequences, to Neoarchean basaltic-andesitic-rhyolitic calc-alkalic and tholeiitic volcanic arc sequences, both with chemical and siliciclastic sedimentary rocks[1]. The 300 m-thick carbonate succession outcropping along the western shore of RLGB consists, from oldest to youngest, of the Balmer, Ball, Slate Bay, Bruce Channel and Confederation Assemblages[2]. The carbonate overlies a thick series of Ball Assemblage ultramafic and mafic volcanic flows capped by approximately 100 m of sandstone. The sandstone is transitional upwards into stromatolitic dolostone[3]. The age of the platform is well constrained by U-Pb zircon dating of rhyolitic lapilli tuff below the carbonates, and a rhyolitic flow above, which places deposition between 2940 ± 2 Ma to 2925 ± 3 Ma[4].

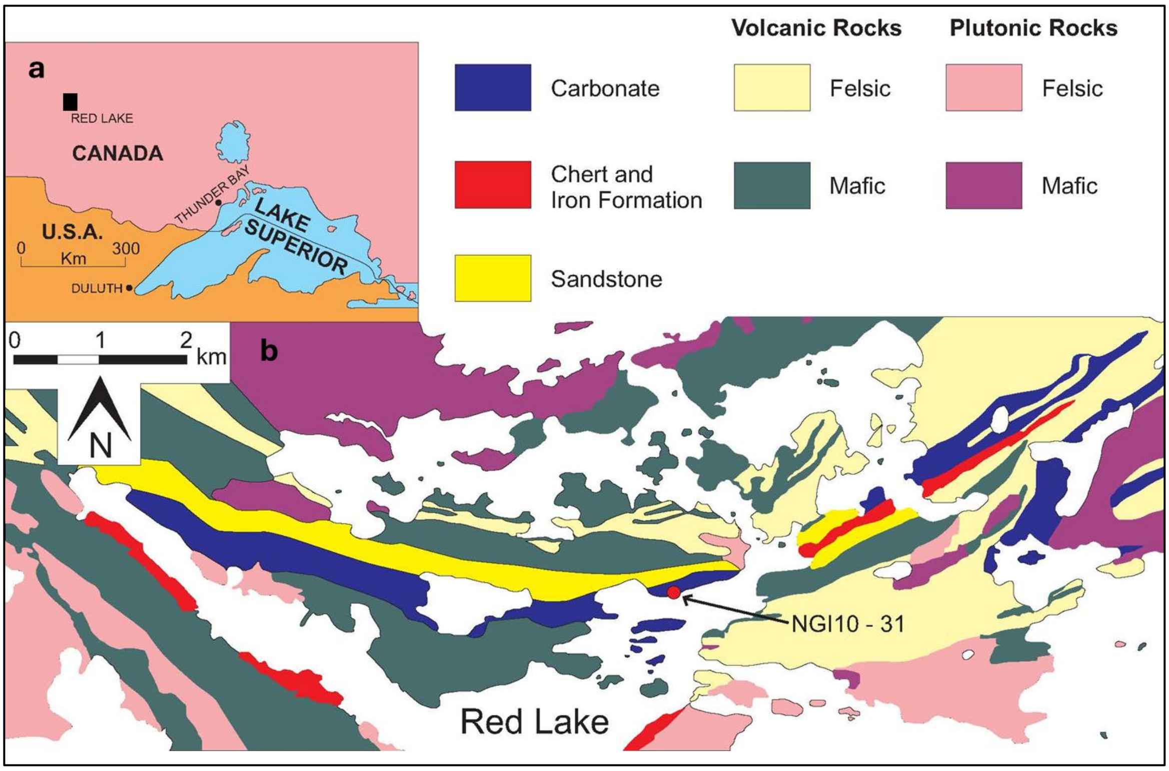

Supplementary Figure 1 NGI10-31 drill core location. **a)** Location of the Red Lake Area indicated by the black square. **b)** Geological map of the Red Lake carbonate platform indicating the location of the drill core NGI10-31.

The western outcrop area of the carbonate succession consists of supratidal coliform crusts in wavy laminated dolostone, and intertidal to very shallow subtidal deposits of stromatolites, layers of small crystal fan fabric, and thin layers of clastic carbonate storm deposits[3]. In deeper water to the east, m-scale limestone mounds composed entirely of large crystal fans are common, as are slides of limestone blocks in iron formation and cm-scale calcite layers intercalated with mm-scale magnetite laminae, representing the edge of the carbonate platform[3,5]. Periods of siliciclastic delivery to the nearshore are represented by layers of sandstone, siltstone and carbonaceous slate ± pyrite. A major flooding event, at approximately 100 m above the base of the carbonate, is denoted by the offshore iron formation, chert and fine-grained siliciclastics transgressing across the carbonate platform[5]. The platform experienced greenschist facies metamorphism and bears no visible or microscopic evidence for secondary or hydrothermal alteration[5].

The NGI10-31 fully cored drill-hole studied here penetrated 75 m of dominantly dolomite underlying the studied interval, with approximately 80 m of the transgressive assemblage consisting of iron formation and siltstone, with minor carbonaceous slate ± sulphide layers. This transition from deposition of carbonate platform sediment to accumulation of offshore lithofacies was probably controlled by the development of transgressive systems track to highstand conditions[6,7]. The iron formation thins, becomes richer in chert and interfingers with dolostone sequences over six km to the west[6] (Supplementary Figure 1b), possibly representing more pronounced parasequence development closer to the shoreline. In this work, we focused on the transgressive sequence formed primarily by oxide iron formation, sulphidic slate, siltstone, and ferruginous chert.

The siliciclastics comprise siltstones and carbonaceous slates that probably represent enhanced incursions of detrital material into the basin. In the Red Lake area, fluvial feeder systems were subject to allocyclic conditions that caused the periodic relocation of their entry point into the marine realm, which commonly occurs through the avulsion of a channel system upstream from its mouth. This would account for periodic changes in the delivery of siliciclastics to this area of the platform, and reflects a temporal rather than spatial control, although the increased influx of siliciclastics may have caused shallowing[5].

The oxide iron formation primarily comprises magnetite interlaminated with chert. Magnetite layering is straight to slightly wavy, parallel, laterally continuous, and commonly has sharp contacts with the chert bands[5]. Similar to the nearby Steep Rock Lake iron deposits[8,9], the deposition of oxide iron formation facies resulted from an offshore, ferrous (Fe^{2+}) iron-rich water mass reacting with a slightly oxygenated water mass close to the redox boundary separating the carbonate shallow shelf

from the further offshore lithofacies[3,5]. This resulted in the accumulation of ferric oxyhydroxides or hydroxides on the bottom, which were eventually transformed into magnetite during diagenesis. The sulphidic slate has a variable thickness (0.5 m to 5 m) and contains either pyrite or pyrrhotite, or a combination of both, and is commonly associated with carbonaceous black slate and interbedded with siltstones and oxide iron formation. The highly reducing environment would have hindered the formation of iron oxides, favouring instead the formation of iron sulphides in conjunction with organic-rich slates. Lastly, successions of white to grey, massive chert of up to 10 m thickness are also present in the NGI10-31 core, which formed through rapid precipitation from low-temperature silica-rich hydrothermal fluids[5].

The alternation between siltstones and the other lithofacies requires that their sedimentation was episodic, and when deposited accumulation rates were much higher than that of the organic-rich mud. Thus, the siltstone layers would represent discrete depositional events of sediment derived from erosion of the landmass washed onto the shallow shelf and extending into the further offshore areas[5,9]. The offshore iron formations, chert and carbonaceous slate were likely deposited in about 200 m of water or less, as there is no extensive development of slump deposits and their transgressions onto the carbonate-dominated portion of the platform indicate they were forming at approximately that depth[5].

Methods

a) Sampling strategy

Core NGI10-31 is located at Thunder Bay University (Canada) and was collected from the Red Lake area, Canada, with coordinates of Easting 416319 and Northing 5653970. The core has a depth of 142 m, and a total of 34 samples were collected between 60 and 140 m depth, of which 30 were used for geochemical analyses. The sampling strategy was designed to ensure the collection of representative samples of all the lithologies present: iron oxide formation, sulphidic slate, ferruginous chert, siltstone and dolostone. Samples were collected approximately every 2.5 m. Portions of the 30 selected samples with no veining or weathering alteration were crushed to a very fine powder using an agate mortar.

b) Iron speciation

Iron speciation analysis was conducted via the well-established sequential extraction procedure[10] at the Géosciences Environnement Toulouse Laboratory (GET), France. Acid-volatile sulphides (AVS) and pyrite Fe (Fe_{Py}) were quantified gravimetrically via a two-step chromium (II) chloride distillation[11],

followed by the precipitation of pyrite sulphur as Ag_2S, in the Cohen Geochemistry Lab at the University of Leeds, UK. The sequential extraction protocol takes ∼0.1 g of powdered sedimentary rock and targets different operationally-defined proportions of iron through three sequential extraction steps: (1) sodium acetate to target iron carbonates (Fe_{Carb}), (2) sodium dithionite for Fe(III) oxides such as goethite and hematite (Fe_{Ox}), and (3) ammonium oxalate for magnetite (Fe_{Mag}). Together, these phases define an Fe pool, which is considered 'highly reactive' (Fe_{HR}) during sedimentation and diagenesis[12], determined as the sum of Fe_{Carb} + Fe_{Ox} + Fe_{Mag} + Fe_{Py}. Iron concentrations (Fe_{Carb}, Fe_{Ox} and Fe_{Mag}) were determined via atomic absorption spectroscopy (ASS) on a Perkin Elmer AAnalyst 400. Replicate analyses of a sample (n = 4) yielded a relative standard deviation (RSD) of 0.4%, 4.3%, 8.3% and 3.1% for Fe_{Carb}, Fe_{Ox}, Fe_{Mag} and Fe_{Py}, respectively. Geochemical data are reported in Supplementary Table 1.

c) Phosphorus phase partitioning

We performed the sequential P extraction scheme[13] adapted for ancient sedimentary rocks[14] at the Géosciences Environnement Toulouse Laboratory (GET), France, on sediment aliquots of ∼100–190 mg. The method targets five operationally-defined sedimentary P pools including iron-bound phosphorus (P_{Fe}), authigenic P (P_{aut}), organic-bound P (P_{org}) and crystalline apatite P (dominantly detrital P; P_{det}). For each extraction step, except for P_{Fe}, P concentrations were determined spectrophotometrically using the molybdate-blue method[15] on a UVisco spectrophotometer at 880 nm. P_{Fe} was measured by inductively coupled plasma–optical emission spectrometry (Horiba Jobin Yvon Expert ICP-OES) due to interference between the extraction chemicals and the molybdate-blue method. We obtained an average recovery of 89% of total P (as determined by bulk extraction; see below), and replicate analyses of a sample (n = 4) gave a relative standard deviation (RSD) of <10% for each step. Geochemical data are reported in Supplementary Table 1.

d) ICP-MS

Bulk sediment digestions were performed at the Center for Astrobiology (CAB), CSIC-INTA, Spain, and at the British Geological Survey (BGS), on ∼50 mg of rock powder using HNO_3/HF/$HClO_4$/H_2O_2. Whole-rock elemental concentrations (major, minor and trace elements) were measured by inductively coupled plasma–mass spectrometry (ICP-MS) on a PerkinElmer Nexion2000C ICP-MS spectrometer. The calibration and optimization of the instrument were performed using a calibration standard solution containing 1 ppb Be, Ce, Fe, ln, Li, Mg, Pb and U to obtain the maximum intensity, as well as oxide/double charge ion lower than 0.03%. Quantitative ICP-MS analysis was performed

using external calibration and internal standardization. The internal standard, which was In, was added to the blank, standards reference materials and komatiite sample solutions. Multi-element standard solutions were used for the external calibration. They consisted of a mixture of diluted solutions prepared from initial mono-element solutions of 1000 mgL^{-1} containing the analytes. The precision, accuracy and memory effects of the analysis were verified by evaluating the recoveries of all the analytes in the quality control multi-element standard solutions. Recovery percentages ranged between 90 and 110% and therefore validated the accuracy and reproducibility of our analysis.

e) Total organic carbon

Total organic carbon analyses through the Rock-Eval pyrolysis technique[16] were performed at the British Geological Survey (BGS, United Kingdom). Aliquots (∼2 g) of crushed sample were weighed into a crucible and analysed in a bulk-flow pyrolysis-flame ionization detector (FID) Rock-Eval 6 pyroanalyser. The instrument was initially calibrated with a standard sample and progressively heated to 600°C in a helium atmosphere. For 3 min, the oven was kept isothermally at 300°C and the free hydrocarbons were volatilized and measured as the S1 peak. The temperature was then increased from 300 to 600°C (at 25°C/min) and hydrocarbons and compounds containing oxygen (CO_2) were produced during the thermal cracking of the insoluble organic matter (kerogen) in the rock (S2 and S3 peaks, respectively), and the residual organic carbon after pyrolysis (S4 peak). The total run time for each sample was approximately 20 min.

TOC was calculated by summing the pyrolyzable carbon (PC) and residual carbon (RC). The PC (generated during the pyrolysis stage) is calculated from the pyrolysis stage-derived S1, S2 and S3 components, while the RC is derived from the oxidation phase by summing the CO (S4CO peak) and CO_2 ($S4CO_2$ peak) generated due to oxidation of organic matter[16]. The precision of the Rock-Eval instrument for TOC was ± 0.1%.

f) Raman spectroscopy

Single Raman spectra of organic matter were obtained in the Geological Spectroscopy Laboratory at UCL with a WITec Confocal Raman Imaging system using a 532 nm laser at up to 1000x magnification. Carbonaceous targets were detected in standard petrological thin sections (30 µm in thickness) using an optical microscope (Olympus BX51) equipped with 4x, 10x, 20x, 50x and 100x objectives. Cosmic ray reduction was applied to all spectra and their backgrounds fitted to a polynomial function and subtracted. Spectra decomposition and peak calculations were calculated by deconvoluting the spectra range from 1090 to 1700 cm^{-1} using a Lorentzian function. After deconvolution, the main key Raman spectral parameters are the peak centre position of five-band

peaks: G-band (∼1594 cm^{-1}), D1-band (∼1350 cm^{-1}), D2-band (∼1620 cm^{-1}), D3-band (∼1510 cm^{-1}), and D4-band (∼1245 cm^{-1}), area, width, and full width at half maximum (FWHM).

To evaluate maximum metamorphic temperatures of organic matter from the Raman spectra we used the geothermometer of reference 17, based on the FWHM values of D1- and D2-bands (FWHM-D1 and FWHM-D2), which show almost linear relations (R^2=0.97 and R^2=0.968) with temperature in the range of approximately 150–400°C[17]. Therefore, these parameters can be used to estimate the metamorphic temperature for low-medium grade rocks with two different equations: T(°C)= −2.15 (FWHM-D1) + 478 and T(°C) = −6.78 (FWHM-D2)+ 535.

Data quality checks

a) Metamorphism

Special caution must be taken when working with ancient sedimentary rocks that have undergone recrystallization and metamorphism. This is because estimations of the reactive P pool may be compromised due to the potential recrystallisation of P_{auth} into well-crystalline apatite, generally extracted as P_{det}[18]. The majority of the Red Lake Greenstone Belt has experienced greenschist to lower amphibolite facies metamorphism[4]. Therefore, it is essential to determine the approximate maximum metamorphic temperatures reached by organic matter locally, in the NGI10-31 core, to evaluate the extent of metamorphic alteration on the samples. In this study, we employed Raman spectroscopy to obtain single-spectrum data from various samples and compared the result with the geothermometer approach described by ref [17]. The results indicate variable temperatures ranging from 340°C to 380°C (Supplementary Figure 2), placing the core samples within the greenschist metamorphic range.

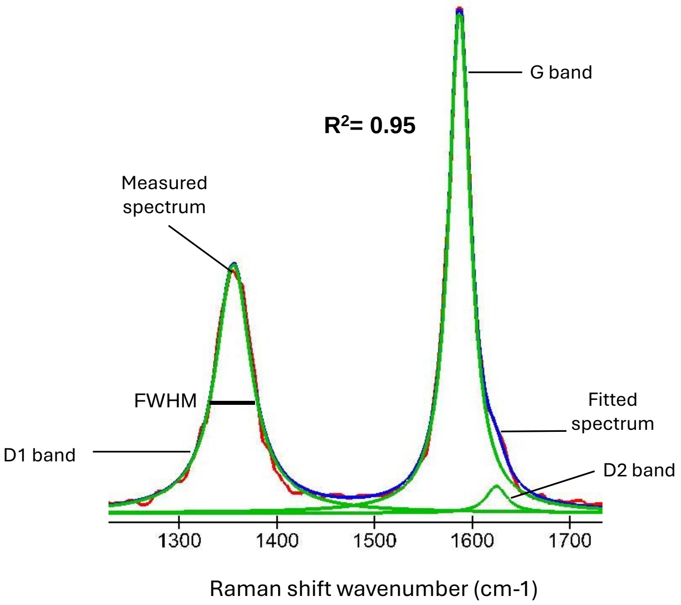

NGI 11	Raman parameters		
	Position	FWHM	Area
G band	1587	26	897
D1 band	1355	43	721
D2 band	1625	25	44.83

T1 (°C)	T2 (°C)
386	366
Average	376

Supplementary Figure 2 Lorentzian peak-fitting of the Raman spectrum and peak metamorphic temperature calculation for the sample NGI 11. The two first-order Raman bands of organic matter at ~1350 cm^{-1} and ~1600 cm^{-1} are represented. Peak decomposition in bands (G, D1, D2) and the full width at half maximum (FWHM) are shown. The coefficient of determination (R^2) compares the original spectrum (red) with the fitted Raman spectrum (blue). Temperatures are calculated with the geothermometer of reference 17.

b) P phase partitioning

A comparison of total P contents from the bulk digestion with the sum of P from the sequential extraction shows a positive correlation (R^2=0.49; Supplementary Figure 3a) and a slope of ~0.95, suggesting that individual P extractions successfully recovered the bulk P content[14]. Note that samples NGI 26 and NGI 29, by contrast, indicate very poor recovery, and we chose to discard these samples to avoid misinterpretation. To evaluate whether the extracted P_{det} truly represents the detrital component, as opposed to recrystallised P_{auth}, we consider the relationship between P_{auth} and P_{det}, which may correlate if P_{det} was in large part a product of P_{auth} recrystallisation[19]. Although we cannot rule out some recrystallization of P_{auth} to P_{det}, the moderate P_{auth} versus P_{det} correlation (R^2 = 0.22) shown in Supplementary Figure 3b, together with the stronger correlation with detrital element Ti (R^2 = 0.48) (Supplementary Figure 3c), indicate that the measured P_{det} content dominantly reflects the actual detrital P input, rather than extensive recrystallization of P_{aut}.

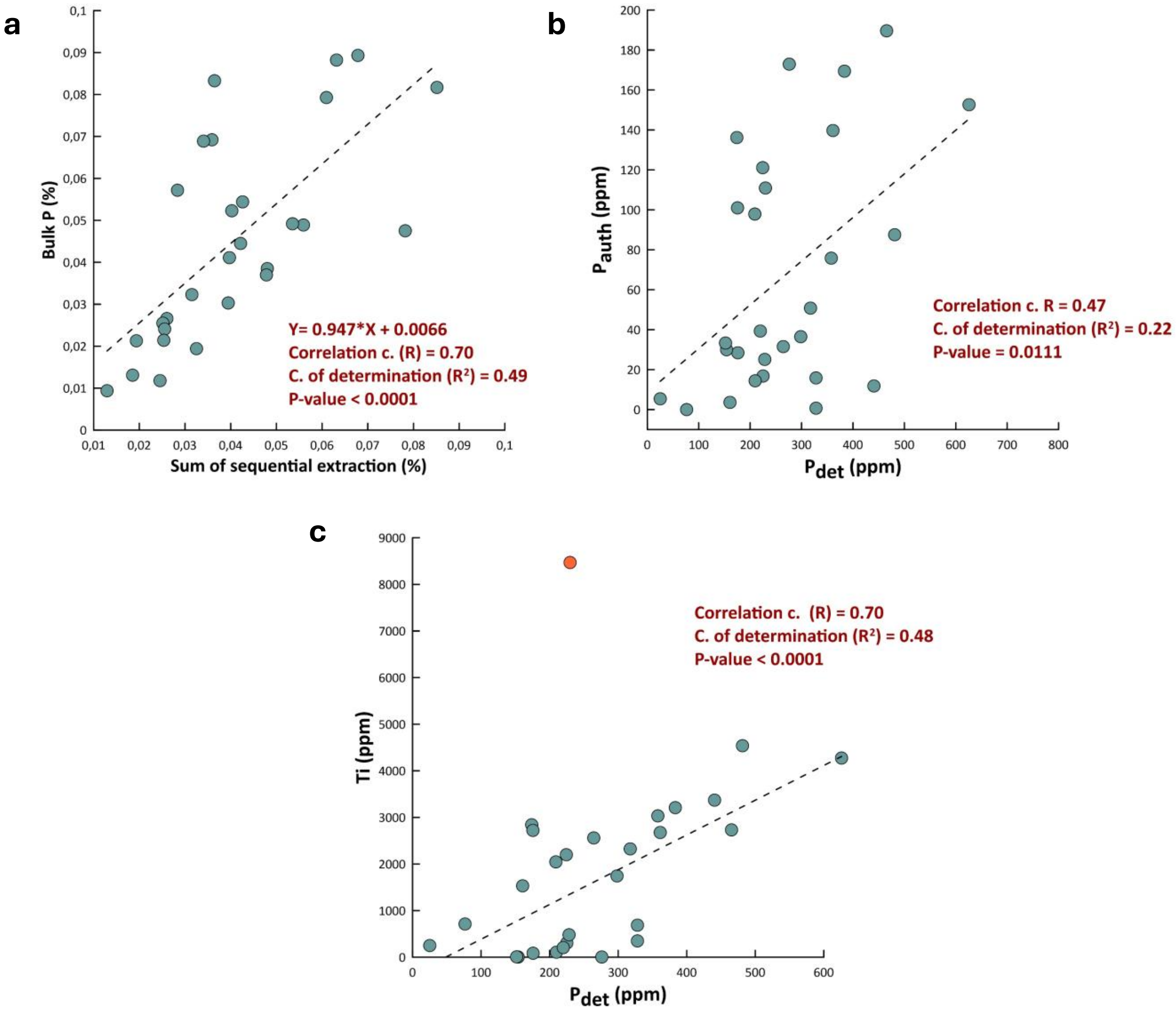

Supplementary Figure 3 Data quality plots and linear regression analysis. a) Sum of P from the sequential extraction vs bulk digestion, showing recovery with the sequential approach. **b) and c)** P phase partitioning quality checks, showing the lack of correlation between P_{auth} and P_{det} (**b**), compared to the correlations between P_{det} and detrital element Ti (**c**), suggesting limited recrystallisation of authigenic apatite into well-crystalline, detrital apatite. Note that in Supplementary Figure 3c, the sample NGI 20 was discarded for the coefficient calculations because of its abnormally high Ti value (~8500 ppm, orange dot).

Supplementary Figure 4

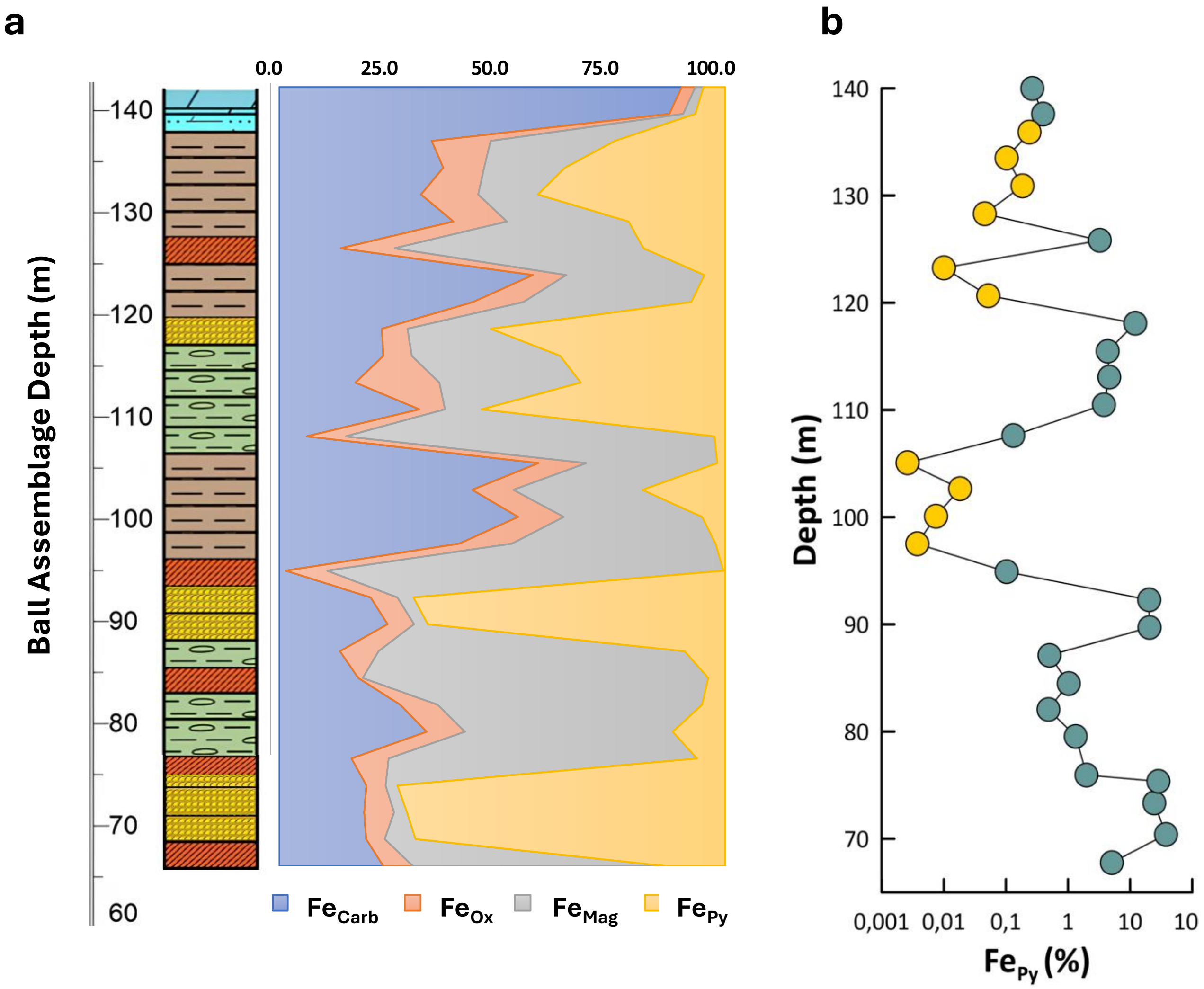

Supplementary Figure 4 NGI10-31 core Fe speciation and Fe_{Py} results. a) Iron speciation results show the proportion of Fe_{Carb}, Fe_{Ox}, Fe_{Mag} and Fe_{Py} within the total Fe pool. **b)** Total Fe_{Py} content. The yellow circles represent the samples deposited under oxic water-column conditions.

References

1. Card, K. D. & Ciesielski, A. Subdivisions of the Superior Province of the Canadian Shield. *Geosci. Canada* **13** (1986).
2. Hollings, P. N. Geochemistry in the Uchi subprovince, northern Superior Province, an evaluation of the geodynamic evolution of the northern margin of the Superior Province ocean basin (1998).
3. McIntyre, T. & Fralick, P. Sedimentology and Geochemistry of the 2930 Ma Red Lake-Wallace Lake Carbonate Platform, Western Superior Province, Canada. *Depos. Rec.* **3**, 258–287 (2017).
4. Corfu, F. & Wallace, H. U–Pb zircon ages for magmatism in the Red Lake greenstone belt, northwestern Ontario. *Can. J. Earth Sci.* **23**, 27–42 (1986).
5. Afroz, M., Fralick, P. W. & Lalonde, S. V. Sedimentology and geochemistry of basinal lithofacies in the Mesoarchean (2.93 Ga) Red Lake carbonate platform, northwest Ontario,

Canada. *Precambrian Res.* **388**, 106996 (2023).
6. Fralick, P. & Pufahl, P. K. Iron formation in Neoarchean deltaic successions and the microbially mediated deposition of transgressive systems tracts. *J. Sediment. Res.* **76**, 1057–1066 (2006).
7. Simonson, B. M. Origin and evolution of large Precambrian iron formations. in *Extreme depositional environments: mega end members in geologic time* (eds. Chan, M. A. & Archer, A. W.) vol. 370 0 (Geological Society of America, 2003).
8. Fralick, P. & Riding, R. Steep Rock Lake: Sedimentology and geochemistry of an Archean carbonate platform. *Earth-Science Rev.* **151**, 132–175 (2015).
9. Riding, R., Fralick, P. & Liang, L. Identification of an Archean marine oxygen oasis. *Precambrian Res.* **251**, 232–237 (2014).
10. Poulton, S. W. & Canfield, D. E. Development of a sequential extraction procedure for iron: Implications for iron partitioning in continentally derived particulates. *Chem. Geol.* **214**, 209–221 (2005).
11. Canfield, D. E., Raiswell, R., Westrich, J. T., Reaves, C. M. & Berner, R. A. The use of chromium reduction in the analysis of reduced inorganic sulfur in sediments and shales. *Chem. Geol.* **54**, 149–155 (1986).
12. Raiswell, R. & Canfield, D. E. Sources of iron for pyrite formation in marine sediments. *Am. J. Sci.* **298**, 219–245 (1998).
13. Ruttenberg, K. C. Development of a sequential extraction method for different forms of phosphorus in marine sediments. *Limnol. Oceanogr.* **37**, 1460–1482 (1992).
14. Thompson, J. *et al.* Development of a modified SEDEX phosphorus speciation method for ancient rocks and modern iron-rich sediments. *Chem. Geol.* **524**, 383–393 (2019).
15. Strickland, J. D. H. & Parsons, T. R. A Practical Handbook of Seawater Analysis. *Fish. Res. Board Canada, Bull.* **167**, 293 (1972).
16. Lafargue, E., Marquis, F. & Pillot, D. Rock-Eval 6 applications in hydrocarbon exploration, production and soil contamination studies. *IFP* **53**, 421–437 (1998).
17. Kouketsu, Y. *et al.* A new approach to develop the Raman carbonaceous material geothermometer for low-grade metamorphism using peak width. *Isl. Arc* **23**, 33–50 (2014).
18. Slomp, C. P., Van Der Gaast, S. J. & Van Raaphorst, W. Phosphorus binding by poorly crystalline iron oxides in North Sea sediments. *Mar. Chem.* **52**, 55–73 (1996).
19. Creveling, J. R. *et al.* Phosphorus sources for phosphatic Cambrian carbonates. *Bull. Geol. Soc. Am.* **126**, 145–163 (2014).
